# The effects PCSO-524®, a patented marine oil lipid and omega-3 PUFA blend derived from the New Zealand green lipped mussel (*Perna canaliculus*), on indirect markers of muscle damage and inflammation after muscle damaging exercise in untrained men: a randomized, placebo controlled trial

**DOI:** 10.1186/s12970-015-0073-z

**Published:** 2015-02-19

**Authors:** Timothy D Mickleborough, Jacob A Sinex, David Platt, Robert F Chapman, Molly Hirt

**Affiliations:** Department of Kinesiology, Human Performance and Exercise Biochemistry Laboratory, School of Public Health-Bloomington, 1025 E. 7th St. SPH 112, Bloomington, Indiana 47401 USA

**Keywords:** Omega-3 fatty acids, Green-lipped mussel oil blend, Muscle damage, DOMS, Eccentric

## Abstract

**Background:**

The purpose of the present study was to evaluate the effects of PCSO-524®, a marine oil lipid and *n*-3 LC PUFA blend, derived from New Zealand green- lipped mussel (*Perna canaliculus*), on markers of muscle damage and inflammation following muscle damaging exercise in untrained men.

**Methods:**

Thirty two untrained male subjects were randomly assigned to consume 1200 mg/d of PCSO- 524® (a green-lipped mussel oil blend) or placebo for 26 d prior to muscle damaging exercise (downhill running), and continued for 96 h following the muscle damaging exercise bout. Blood markers of muscle damage (skeletal muscle slow troponin I, sTnI; myoglobin, Mb; creatine kinase, CK), and inflammation (tumor necrosis factor, TNF-α), and functional measures of muscle damage (delayed onset muscle soreness, DOMS; pressure pain threshold, PPT; knee extensor joint range of motion, ROM; isometric torque, MVC) were assessed pre- supplementation (baseline), and multiple time points post-supplementation (before and after muscle damaging exercise). At baseline and 24 h following muscle damaging exercise peripheral fatigue was assessed via changes in potentiated quadriceps twitch force (∆Q_tw,pot_) from pre- to post-exhaustive cycling ergometer test in response to supra-maximal femoral nerve stimulation.

**Results:**

Compared to placebo, supplementation with the green-lipped mussel oil blend significantly attenuated (p < 0.05) sTnI and TNF-α at 2, 24, 48, 72 and 96 h., Mb at 24, 48, 72, 96 h., and CK-MM at all-time points following muscle damaging exercise, significantly reduced (p < 0.05) DOMS at 72 and 96 h post-muscle damaging exercise, and resulted in significantly less strength loss (MVC) and provided a protective effect against joint ROM loss at 96 h post- muscle damaging exercise. At 24 h after muscle damaging exercise perceived pain was significantly greater (p < 0.05) compared to baseline in the placebo group only. Following muscle damaging exercise ∆Q_tw,pot_ was significantly less (p < 0.05) on the green-lipped mussel oil blend compared to placebo.

**Conclusion:**

Supplementation with a marine oil lipid and *n*-3 LC PUFA blend (PCSO-524®), derived from the New Zealand green lipped mussel, may represent a useful therapeutic agent for attenuating muscle damage and inflammation following muscle damaging exercise.

## Introduction

Exercise-induced muscle damage (EIMD) can be caused by eccentric type or unaccustomed (novel) exercise, and results in decrements in muscle force production, development of delayed-onset muscle soreness (DOMS) and swelling, rise in passive tension, and an increase in blood intramuscular proteins [1]. Delayed-onset muscle soreness is generally considered a hallmark sign of EIMD [2], and it is thought that DOMS is partially related to direct muscle fiber damage, and its magnitude appears to vary with the type, duration and intensity of exercise [3]. The inflammatory response to EIMD results in the release into blood of reactive species from both neutrophils and macrophages, and an array of cytokines from the injured muscle including tumor necrosis factor (TNF)-α, interleukin (IL)-1β and IL-6, which contribute to a low-grade systemic inflammation and oxidative stress [4]. The pro- inflammatory and pro-oxidant response can provoke secondary tissue damage [5], thus prolonging the regenerative process, which is generally characterized by a restoration of muscle strength and resolution of inflammation [5].

Exercise-induced muscle damage and DOMS can potentially hinder performance in activities ranging from basic physical activity to athletic training and competition. There are a number of strategies that have been used to attenuate EIMD and DOMS such as anti-inflammatory medication, cryotherapy, massage, stretching, hyperbaric oxygen, homeopathy, ultrasound, rest, light exercise and electrotherapeutic modalities [3]. The use of non-steroidal anti- inflammatory drugs (NSAIDs) and continued exercise appear to be the most commonly used methods to treat DOMS [1]. However, while the use of NSAIDs has been shown to decrease perceived muscle soreness and pain associated with DOMS, they fail to impact the length or degree of muscle weakness [6], may be detrimental to muscle cell repair and adaptation by decreasing satellite cell activity [7], and have been shown to suppress the protein synthesis response in skeletal muscle after eccentric resistance exercise [8]. Due to the fact that there appears to be no completely effective treatment for preventing/reducing EIMD and treating DOMS [1,6], the use of complimentary therapy, in particular nutraceuticals (e.g. tart cherry juice [9], curcumin [10], and quercetin [11]) that possess anti-inflammatory properties and have the potential to attenuate EIMD-induced oxidative stress, have become of interest [1].

One class of nutrients that appears to possess both anti-inflammatory and anti-oxidant properties are the long-chain omega (*n*)-3 long chain polyunsaturated fatty acids (LC-PUFA), such as eicosapentaeoic acid (EPA; 20:5 n-3)) and docosahexaenoic acid (DHA; 22:5 n-3), found in fish oil. Numerous studies have shown that *n*-3 LC-PUFA administered at doses greater than one gram per day have beneficial actions in many inflammatory diseases, cancer, and human health in general [12], and that *n*-3 LC-PUFA may act as important energetic molecules that can modulate immune, inflammatory, and oxidative stress responses to exercise [13]. A small number of studies have sought to evaluate whether fish oil supplementation can reduce the degree of skeletal muscle injury, inflammation and oxidative stress following eccentric exercise [13]. Although more studies have demonstrated a positive effect of *n*-3 LC-PUFA in relation to ameliorating muscle damage, DOMS, inflammation, and oxidative stress following eccentric exercise [14-20], some investigations have shown no effect [14,21]. It is likely that the diversity in testing protocols, supplementation dosage and duration, subject population, timing of measurements and selection of biomarkers contribute to the discrepancies in the findings between studies. However, it is possible that different forms of marine oils may have varying effects on these responses, since these oils contain a variety of lipid mediators as well as a different amount of *n*-3 LC-PUFA.

PCSO-524® (Lyprinol®/Omega XL®) is a nutritional supplement comprising of a patented extract of a very condensed form of stabilized marine lipids from the New Zealand green lipped mussel, *Perna canaliculus*, combined with olive oil and vitamin E [22,23]. This marine oil lipid and *n*-3 PUFA blend is a multifarious mixture of sterol esters, sterols, polar lipids, triglycerides, EPA and DHA (split between the triacylglycerol and polar lipid classes), and free fatty acids [24], and has been shown to exert its anti-inflammatory effects via furan fatty acids [25], and inhibition of cyclooxygenase (COX)-2 and 5-lipoxyeganse (LOX) pathways for the metabolism of arachidonic acid, thereby leading to a subsequent reduction in pro-inflammatory leukotriene, prostaglandin, and cytokine production from inflammatory cells [22,26]. A number of human and animal studies have shown that the green-lipped mussel oil blend may have beneficial effects in treating inflammatory diseases such as osteoarthritis, rheumatoid arthritis, inflammatory bowel disease, asthma [22], and exercise- induced bronchoconstriction [27]. These preliminary findings support the potential for supplementation with the green-lipped mussel oil blend in order to attenuate muscle damage and inflammation that can occur following muscle damaging exercise.

Therefore, the primary aim of the present study was to evaluate the effects of supplementation with a green-lipped mussel oil blend on indirect markers of muscle damage, inflammation, and quadriceps fatigue following muscle damaging exercise in untrained men. We hypothesized that supplementation with a green-lipped mussel oil blend, compared to placebo, would significantly reduce blood markers of muscle damage and inflammation, and modulate quadriceps fatigue and functional measures of muscle damage following downhill running designed to induce muscle damage in untrained men.

## Methods

### Subjects

Forty untrained males volunteered to participate in the study, and of these thirty-two subjects (aged 22.0 ± 2 y, height 176.3 ± 7.0 cm, body mass 70.8 ± 9.8 kg, maximal oxygen consumption (VO_2peak_) 46.0 ± 6.1 mL∙kg^−1^∙min^−1^) completed the study. Reasons for the non- inclusion of eight subject data sets in the final statistical analysis were (1) subjects failing to show up at testing sessions (incomplete data; n = 3), (2) inability of the investigators to obtain a blood sample (incomplete data; n = 3), and (3) identification of erroneous recordings of data (n = 2). Subjects were classified as ‘untrained’ if they exercised less than three times per wk for less than 30 min during each session. Subjects were excluded if they had a history of significant pain in hips or knees, had participated in a strength training program within 60 d prior to study participation, or regularly used nutritional supplements and over-the-counter and prescription anti-inflammatory medication. All subjects were screened for coronary artery disease risks factors as per the American College of Sports Medicine guidelines [28]. Subjects were instructed to refrain from downhill running, stair running, resistance training, plyometric or other mode of exercise that could potentially cause muscle damage, and to refrain from modifying their exercise habits during the course of the study. Adherence to these instructions was confirmed at each visit to the laboratory. The study was approved by the Indiana University Institutional Review Board for Human Subjects, and written informed consent for all subjects was obtained prior to participation in the study.

### Study design

The study was conducted as a randomized, double-blind, placebo-controlled parallel group trial over 30 days. This design was chosen over a crossover design in order to avoid the repeated-bout effect acting as a confounding variable [1]. Subjects were randomly assigned to either a green-lipped mussel oil blend (PCSO-524®) supplementation group (n = 16) or a placebo group (n = 16). Supplementation with the green-lipped mussel oil blend and placebo began 26 days before an eccentric exercise bout (downhill running, designed to induce muscle damage) and continued for 4 days following the muscle damaging exercise bout. An activity diary and food frequency questionnaire was completed by each subject during the course of the study.

#### Pre-supplementation measures

After subjects provided written informed consent for participation and the investigators explained the study protocol, all subjects underwent an exhaustive 20-min cycle ergometer familiarization test (T1 day −21), followed one week later (T2 day −14) by an incremental treadmill load test of their maximal oxygen uptake (VO_2max_), in order to determine the intensity (70% VO_2peak_) the subjects will exercise at for the eccentric exercise test. One week (T3 day −7) following the VO_2peak_ test an initial (baseline) blood draw was taken in order to measure baseline blood markers of muscle damage, inflammation and DNA oxidative stress, along with baseline functional measures of muscle damage [i.e. isometric torque (MVC), knee flexion (joint range of motion), limb girth (swelling), muscle soreness, and muscle pain], which were followed one week later (T4 day 0) by measures of quadriceps muscle fatigue (quadriceps twitch force measured via femoral magnetic nerve stimulation before and after a 20-min exhaustive cycle ergometer test).

#### Post-supplementation measures

Following the 26 days of supplementation a venous blood draw was taken and functional measures of muscle damage conducted (T5 day 26), which was directly followed by the downhill running protocol specifically designed to induce muscle damage [29,30] (T6 day 26). Immediately (T7 day 26) and 2 h following the muscle damaging exercise a venous blood draw was taken (T8 day 26), followed by additional blood draws and functional measures of muscle damage at 24 h (T9 day 27), 48 h (T10 day 28), 72 h (T11 day 29) and 96 h (T12 day 30) post-muscle damaging exercise. Quadriceps muscle fatigue was measured 24 h following the muscle damaging exercise bout (T8 day 27). The timing of this measurement was chosen in order to correspond with expected decrements in muscle strength, range of motion and significant increases in swelling, tenderness and soreness. On testing days T3 day −7, T5 day 26, T9 day 27, T10 day 28, T11 day 29 and T12 day 30 the sequence of procedures comprised the following order: blood draws, DOMS, range of motion, pressure pain threshold, thigh girth (swelling) and isometric torque (MVC). On testing T9 day 27 only, subjects underwent the protocol for the measurement of quadriceps muscle fatigue before and after the 20-min exhaustive cycle ergometer trial.

### Supplementation

Subjects ingested either 8 capsules per d of PCSO-524® (Lyprinol®/Omega XL®; Pharmalink International Ltd, Hong Kong) (n = 16), which equaled 800 mg olive oil, 400 mg lipid extract (~58 mg EPA and 44 mg DHA) and 1.8 mg vitamin E (d-alpha-tocopherol) or 8 placebo capsules containing olive oil (1200 mg olive oil) (n = 16) for 30 d. Each PCSO-524® capsule contains 50 mg lipid extract (fatty acids), 7.3 mg (14%) EPA, 5.5 mg (11%) DHA, 100 mg olive oil and 0.225 mg vitamin E, and 1 placebo capsule contains 150 mg olive oil. The active PCSO-524® capsules containing the green-lipped mussel oil blend were identical in size, color, texture and taste to their respective placebo counterpart. Product specification was provided to the investigators by the trial sponsor (Pharmalink). Cawthron Laboratories (Nelson, NZ), an independent laboratory, completed the fatty acid analysis of the raw material, and Chemisches Labor (Hannover, Germany) conducted the final fatty acid testing of the finished PCSO-524 ® capsuled product. Alpha laboratories (Auckland, NZ) conduced the fatty acid analysis on the placebo (olive oil) capsules. While Table 1 presents the fatty acid analysis conducted on the PCSO-524® (Batch No. A6530-01) and placebo (Batch No. 7820) capsules used in the present study, a detailed fatty acid analysis of the PCSO-524® and placebo capsules has been published elsewhere [31,32]. Wolyniak et al. [23] have shown that the ‘lipid extract’ portion of the green-lipped mussel oil blend contains up to 91 fatty acids (including EPA and DHA). Of the 91 fatty acids reported [23], 16 represented more than 1% of the total FA. In decreasing order of abundance, these were EPA, C16:0 (Palmitic acid), DHA, C14:0 (Myristic acid), C16:1n-7 (Palmitoleic acid), C18:0 (Steroic acid), C18:1n-5, C18:4n-3 (Stearidonic acid), C18:2n-6 (Linoleic acid), C20:4n-6 (Arachidonic acid), C18:3n- 3 (Alpha-linoleic acid), C16:1n-5, C20:1n-9 (Eicosenoic acid), C18:1n-9 (Oleic acid), C15:0 (Pentadecanoic acid), and C16:1n-9 (7-(hexadecenoic acid). PCSO-524® is a natural product subject to variations in the New Zealand Marlborough Sounds ecosystems. Values in the specification of this organic compound can vary according to season and climate temperatures, and therefore, during the manufacturing process a variance of +/− 10% in the saturated, monounsaturated and PUFA composition of PCSO-524® is deemed acceptable.

**Table 1:** **Fatty acid composition (%) of PCSO-524®, a marine oil extract of the New Zealand green-lipped mussel (** ***Perna canaliculus*** ) * and placebo (olive oil) ** capsules

**FA nomenclature**	**Fatty acid name**	**PCSO-524®capsules (Weight, %)**	**Placebo (Olive oil) capsules (Weight, %)**
14:0	Myristic acid	1.7	
16:0	Palmitic acid	13.4	9.2
16:1	Palmitoleic acid	3.6	3.0
18:0	Stearic acid	3.6	3.5
18:1	Oleic acid	58.2	81.0
18:2n-6	Linoleic acid	5.7	2.6
18:3n-3	Alpha-linolenic acid	0.9	0.4
18:4n-3	Octadecatetraenoic acid	1.0	
20:0	Arachidic acid	0.4	0.3
20:1	Eicosamonoenoic acid	0.7	
20:4n-6	Arachidonic acid	0.1	
20:4n-3	Eicosatetraenoic acid	0.3	
20:5n-3	Eicosapentaenoic acid	5.8	
22:5n-3	Docosapentaenoic acid	0.3	
22:6n-3	Docosahexaenoic acid	3.0	
Others		1.3	

*Batch number: A6530-01. **Batch number: 7820. FA nomenclature: number of carbon atoms (chain length), number of double bonds, and position of the last double bond from the methyl (omega) end.

### Experimental measures

#### Peak aerobic exercise capacity (VO_2peak_)

Subjects performed a peak aerobic exercise capacity test, adapted from a previously published protocol from our laboratory [33], on a motor driven treadmill (Model 18–60, Quinton, Seattle, WA), while fitted with a heart rate monitor (Polar Electro Inc., Lake Success, NY) and breathing mask (7450 Series V2, Hans Rudolph, Shawnee, KS USA). The protocol started with a warm-up period of 5 min, in which subjects chose a comfortable running speed that they would be expected to be able to continue on a level treadmill for 15 min; selected speeds ranged from 7.2 – 13.8 km/h. After 5 min of seated rest, the exercise portion of the test began with each subject running at 0% grade at a speed of 1.6 k/h less than the selected (warm-up) speed for 2 min. Following the initial stage, the speed was increased to the predetermined speed. After 3 min, the slope of the treadmill was increased to 4% for 3 min, and then increased an additional 2% every 3 min until volitional exhaustion or valid test criteria were met. Ventilatory and metabolic data were collected using open-circuit, indirect calorimetry. Dried expired gases were sampled at a rate of 300 mL∙min^−1^ for fractional concentrations of O_2_ and CO_2_ using an Applied Electrochemistry S-3A oxygen analyzer and a CD-3A carbon dioxide analyzer (Ametek, Thermox Instruments, Pittsburgh, PA). Inspired ventilation was measured with a pneumotachometer (Hans Rudolph 3813).

#### Eccentric muscle damaging exercise

All subjects performed a 20-min downhill running bout on a motorized treadmill (A.R. Young Company, Indianapolis) modified to run in reverse at a −16% grade, which is a protocol that has previously been shown to elicit a significant degree of muscle damage following downhill running [29,30]. Once the test commenced subjects were not allowed to stop, and treadmill speed was adjusted so that the subjects maintained a heart rate that corresponded to 70% VO_2max_. It has been shown that downhill running is effective in causing skeletal muscle damage, symptoms of DOMS, and loss of muscle force [34].

#### Delayed onset muscle soreness and pain threshold

Lower limb soreness was assessed using a visual analog (numeric) rating pain scale with “no soreness” indicated at one end (score 0) and “unbearably painful” at the other (score 10) Subjects stood with hands on hips and feet approximately shoulder width apart. The subject was asked to squat down to 90° (internal angle), rise to the start position and then indicate on the numeric scale the soreness felt in the lower limbs.

The pressure pain threshold was measured at five specific sites on the quadriceps with a digital algometer (Force One, Wagner Instruments, Greenwich, CT.) to quantify muscle tenderness. The same investigator performed all measurements throughout the study. Specific sites for assessment were determined using established literature and landmarks [32], involving two anatomical points (anterior superior iliac spine (ASIS) and superior pole of the patella (SPP). All measurements were taken on the right side with the subject in the supine position. A longitudinal axis was created between the ASIS and the SPP from which the sites were marked with a permanent marker to ensure accuracy at each time point. The measured sites were: 15 cm distal to the ASIS, 4 cm proximal to the SPP, midpoint of the ASIS and SPP along the axis, then 2 cm lateral and 2 cm medial of this midpoint. Subjects were instructed to let the investigator know when the pressure transformed into pain at which point the amount of force was recorded in newtons (N).

#### Range of motion (knee flexion), swelling (thigh girth) and isometric strength (torque)

Range of motion has been shown to be an accurate method of determining the extent of muscle damage [35]. Subjects were instructed to lay prone on a massage table with both knees fully extended. Subjects flexed their left knee with no assistance from the investigator, and the angle measured with a goniometer (Prestige Medical, Northridge, CA) using universal landmarks (lateral epicondyle of the femur, lateral malleolus and greater trochanter) that were marked with a permanent marker to ensure consistency on subsequent measures. Three measurements were averaged and reported in degrees. This method for a assessing ROM has been validated previously [36].

In order to determine the presence of swelling/edema within a muscle thigh circumference was assessed at the midpoint of the ASIS and SPP of the right leg with an anthropometric tape (Idass, Glastonbury, UK). Subjects were standing fully relaxed in the anatomical position. Subjects were instructed to put all their weight on the opposite leg and 3 measurements were taken. Measurement sites were marked to ensure consistent measurements and the average was reported.

Isometric torque was assessed at a knee angle of 80° using previously described protocol [37]. Subjects were seated in a chair, secured with a belt across the legs and chest, and their left leg secured to a force transducer (Model Z Tension Load Cell, Dillon, Fairmont, MN) with a non-compliant strap. Subjects were familiarized with the equipment by performing three warm-up contractions (two submaximal, 1 maximal) separated by 10 seconds of rest, followed by a 5 min recovery. After the recovery period, subjects performed 3 maximum voluntary contractions (MVCs) of the quadriceps, interspersed with a 10 s recovery interval between contractions. The highest peak torque from the 3 contractions was recorded. Subjects were verbally encouraged during the contractions to produce a maximum effort.

#### Quadriceps muscle fatigue

Potentiated quadriceps twitch force was measured in the subject’s left leg to quantify an index of muscle fatigue following a 20 min exhaustive cycle ergometer test. Subjects lay semirecumbent on a table with a left knee joint angle of 90 degrees. The subject’s ankle was wrapped in a non-compliant strap, placed just superior to the ankle malleoli. The strap was attached to a calibrated load cell (Model Z Tension Load Cell, Dillon, Fairmont, MN) for the measurement of force connected to a custom amplifier (Hector Engineering Co. Inc., Ellettsville, IN). A magnetic stimulator (Magstim 200, Magstim, Whitland, UK) connected to a double 70 mm coil was used to stimulate the femoral nerve, causing an involuntary contraction of the quadriceps muscle. Nerve stimulation followed two protocols, which have been described previously [38].

#### Assessment of maximal nerve stimulation

Prior to the exhaustive 20 min cycle ergometer test, a series of single twitches were obtained at varying levels of stimulator intensity (80%, 85%, 90%, 95%, and 100% of maximal stimulator power output) to determine when supramaximal stimulation had been reached. The position of the stimulator coil was placed over the femoral triangle and adjusted to determine an acceptable location for each subject. Stimulator placement was determined to be acceptable when repeatable and measurable quadriceps contractions were obtained. Stimulator placement was marked on the subject’s skin with an indelible marker to insure repeatability of the location and measurement. Typical stimulator output required to achieve supramaximal stimulation has been found to be a mean of approximately 83% of stimulator output [39].

#### Exhaustive 20 min cycle ergometer test

Following a 5 min warm up at a self-selected intensity, subjects completed a 20 min exercise task on a cycle ergometer (Velotron, RacerMate Inc., Seattle, Washington, USA). Subjects were allowed to change resistance freely and were asked to complete the furthest possible distance, and to achieve the highest possible power output, during the 20 min ergometer test.

#### Assessment of fatigue

Prior to and immediately following the exhaustive cycle ergometer test, an assessment of quadriceps twitch force (Q_tw,pot_) was performed. Twitch force prior to the 20 min cycle ergometer test was used as a baseline for twitch force obtained after the time trial. The assessment of fatigue protocol consisted of six repetitions of potentiation and magnetic stimulation with 30 s of rest between repetitions. For each repetition, subjects performed a maximal voluntary isometric contraction (MVC) of the quadriceps muscle for 5 s. At the end of the 5 s MVC, the subject received a supra-maximal magnetic stimulation of the femoral nerve, and a second stimulation after 5 seconds of rest [40]. The force produced during the second twitch of each repetition was recorded as Q_tw,pot_. Force values from the first two repetitions were discarded based on previous findings that the degree of potentiation is smaller after the first two measurements [38]. Force values from the final four repetitions were averaged to produce a Q_tw,pot_ force value for each trial.

### Blood sampling and analysis

All blood draws were taken from the antecubital vein and collected into 10 ml plain Vacutainer® clot tubes (PulmoLab, Porter Ranch, CA). The tubes were gently inverted five times after collection to mix the clot activator with blood, and then placed on ice for at least 30 minutes before centrifugation (Allegra ™ X-22R Centrifuge, Beckman Coulter, Inc., Brea, CA) at 20°C at 3000 RPM for 15 min. Serum was removed after spinning and allocated to storage tubes and stored immediately at −80°C until later analysis of muscle damage, inflammation and oxidative stress markers using enzyme immunoassay techniques [Powerwave XS™ Spectrophotometer (Bio-Tek Instruments, Winooski, VT)] according to manufacturer’s instructions.

#### Skeletal and cardiac muscle damage

Creatine kinase, muscle (CK-MM) was assessed using a sandwich enzyme linked immunoassay test (sensitivity: 12.8 U/L; Detection range: 31.2 – 2,000 U/L. Intra-assay precision: CV < 10%; Inter-assay precision: CV < 12% as per the manufacturer’s (Caltag Medsystems Ltd, Milton Keynes, UK) protocol. Skeletal muscle slow troponin I (sTnI) was assessed using a sandwich enzyme linked immunoassay test (Minimum detectable concentration typically 5.4 pg/ml; Detection range: 15.6-1,000 pg/ml; Intra-assay precision: CV < 10%; Inter-assay precision: CV < 12% as per the manufacturer’s (USCN Life Science Inc., Hubei, Peoples Republic of China) protocol. Myoglobin (Mb) was assessed using an enzyme-linked immunoassay test (minimum detectable concentration: 5.0 ng/ml; sensitivity: 25 ng/ml; Detection range: 25.0-1,000 ng/ml) following the manufacturer’s recommendations (Calbiotech, Spring Valley, CA, USA). Cardiac troponin I (CTnI) was analyzed a using sandwich enzyme-linked immunoassay test (minimum detectable concentration: 0.45 ng/ml; Detection range: 0.48-5.0 ng/ml; inter-assay precision: <10%) as per the manufacturer’s (Abnova, Taipei, Taiwan) recommendations. Human heart fatty acid binding protein (hFABP) was assessed using a sandwich enzyme linked immunoassay test (minimum detectable concentration: 156 pg/ml; Detection range: 312 – 20,000 pg/ml. Intra-assay precision: CV < 4-6%; Inter-assay precision: CV < 8-10%) following the manufacturer’s (Innovative Research, Novi, MI, USA) recommendations.

#### Inflammatory and DNA oxidative stress markers

Tumor necrosis factor (TNF)-α was assessed using a sandwich enzyme linked immunoassay test (sensitivity: 1.7 pg/ml; Detection range: 15.6 – 1,000 pg/ml. Intra-assay precision: CV < 4.4%; Inter-assay precision: CV < 7.5% as per the manufacturer’s (Invitrogen Corp., Camarillo, CA, USA) protocol. 8-Oxo-2'-deoxyguanosine (8-OhdG) was analyzed using a competitive enzyme linked immunoassay test (Detection range: 100 pg/ml – 20 ng/ml) as per the manufacturer’s (Cell Biolabs Inc., San Diego, CA, USA) recommendations.

### Nutrient intake and compliance

All subjects were given an activity diary to record frequency, mode and duration of exercise. Nutrient intake was monitored to ensure that dietary factors would do not change through the course of the study, and potentially affect the dependent measures. Nutrient data was collected using the GSEL food frequency questionnaire (FFQ) developed by the Nutrition Assessment Shared Resource (NASR) of Fred Hutchinson Cancer Research Center. Subjects completed the GSEL version of the questionnaire before supplementation and at the end of the 30 day supplementation period. Analysis of GSEL for nutrient intake was conducted at the Fred Hutchinson Cancer Research Center. Nutrients of interest obtained from the GSEL analysis included macronutrient composition, antioxidants (α-tocopherol, β-carotene, lycopene, Vitamin C), certain minerals (magnesium, sodium, zinc), and types of dietary fatty acids (omega-3, total polyunsaturated fatty acids, saturated fatty acids). While the FFQ has been shown to be valid and reliable in the collection of dietary data [41], we acknowledge that diet may act as confounding factor, since it was not directly controlled for in our study. Adherence to the treatment regimen was monitored by asking the subjects to document the dose of capsules consumed daily and to return any unused capsules. For the purpose of the present study a compliance of ≥90% was considered acceptable.

### Data analysis

The data were analyzed using a two-way (group, 2; time, 6–8) split-plot repeated measures ANOVA using SPSS version 20.0 (IBM Corporation, Chicago, IL, USA) statistical software. The data was assessed for normality using the Kolmogorov–Smirnov test, and Levene’s test was used to test for homogeneity of variance between groups. Mauchly’s test was be conducted to determine whether sphericity is violated. If sphericity is violated, the repeated-measures ANOVA was corrected using the Greenhouse–Geiser correction factor. A fisher’s protected least-square difference post-hoc test was used *a priori* to determine differences in dependent measures within and between groups. Statistical significance was set at p ≤ 0.05. Data are expressed as mean ± SD.

To determine an appropriate sample size for present study, a post-hoc power analysis of existing literature was conducted using G*Power version 3.0.5 (Universität Kiel, Germany). Based on two studies [19,20] investigating the efficacy of *n*-3 LC-PUFA on DOMS, and blood markers of muscle damage and inflammation following eccentric exercise, achieving an experiment-wise error rate of 0.05 required 15 subjects within each treatment group. In these studies, Tartibian et al. [19,20] has shown that ingestion of *n*-3 LC-PUFA (n = 9–15) for 30 days compared to placebo/control (n = 9–15) significantly reduced inflammatory markers, and perceived pain and symptoms, following eccentric exercise, with effect sizes ranging from 0.64-0.75 for a study power of 0.82 and 0.84 respectively.

## Results

### Subject characteristics

There were no significant differences (p > 0.05) for age, height, BMI, VO_2max_ (L) and VO_2peak_ (mL/kg/min) between the green-lipped mussel oil blend (PCSO-524™) and placebo group (Table 2). However, body mass (kg) was significantly different (p < 0.05) between groups.

**Table 2:** **Subjects**’ **baseline characteristics**

	**Green**-**lipped mussel oil blend** ** (n = ** **16)**	**Placebo** **(n = ** **16)**	**p**-**value**
Age (years)	21.7 + 1.7	21.5 + 2.4	0.803
Height (cm)	178.1 + 5.8	174.2 + 6.7	0.091
Body mass (kg)	74.8 + 8.8	66.6 + 9.7	0.018*
BMI (kg/m^2^)	23.6 + 2.9	21.9 + 2.8	0.102
^VO^2peak ^(L)^	3.4 + 0.5	3.0 + 0.6	0.073
^VO^2peak ^(ml/kg/min)^	46.4 + 6.2	45.6 + 6.1	0.732

*Significantly different (p < 0.05) between groups. BMI, body mass index. VO2peak, peak oxygen consumption. Values are expressed as mean ± SD.

### Delayed onset muscle soreness and pain threshold

Muscle soreness significantly increased (p < 0.05) in both groups after the muscle damaging exercise protocol, peaking between 24 and 48 h and declining toward baseline at 72 and 96 h (Table 3). Significant effects for time were found in the green-lipped mussel oil blend group (p < 0.001) and placebo group (p < 0.001). Post-hoc pairwise comparisons between groups at each time point revealed significantly lower DOMS in the treatment group, compared to placebo, at 72 h [p = 0.027; mean difference (∆), 1.25 ± 2.41; 95% CI (difference of means), 0.08 to 2.52] and 96 h (p = 0.037; ∆, 1.25 ± 1.95; 95% CI, 0.13 to 2.63%) following muscle damaging exercise. However, there were no significant differences (p > 0.05) between groups prior to supplementation (baseline) and following supplementation (before, and at 24 and 48 h following muscle damaging exercise).

**Table 3:** **Effect of supplementation on functional measures of muscle damage and fatigue following eccentric exercise**

**Variables/** **Groups**	**Pre**- **supplementation**	**Post**-**supplementation**				
	**(Baseline)**	**Before muscle damaging exercise**	**24 h after muscle damaging exercise**	**48 h after muscle damaging exercise**	**72 h after muscle damaging exercise**	**96 h after muscle damaging exercise**
DOMS (arbitrary units)						
Green-lipped mussel oil blend	2.3 ± 1.7	2.0 ± 2.0	4.9 ± 2.7^¥,#^	4.6 ± 2.2^¥^	2.7 ± 1.7^#^	1.8 ± 1.6^#^
Placebo	1.9 ± 1.6	1.4 ± 1.5	4.4 ± 2.1^¥, #^	4.8 ± 1.8^¥^	3.9 ± 1.8^¥,#^	3.0 ± 2.2^¥,#^
p-value*	0.700	0.563	0.847	0.441	0.029*	0.037*
Pressure Pain Threshold (% Δ from baseline)						
Green-lipped mussel oil blend	-	−0.08 ± 0.32%	−0.13 ± 0.26%	−0.12 ± 0.28%	−0.07 ± 0.34%	−0.01 ± 0.34%
Placebo	-	−0.03 ± 0.35%	−0.15 ± 0.27%^#^	−0.10 ± 0.39%	−0.07 ± 0.41%	0.01 ± 0.50%
p-value*	-	0.643	0.844	0.824	0.978	0.896
Knee Flexion Range of Motion (degrees)						
Green-lipped mussel oil blend	47.5 ± 6.1	46.7 ± 5.0	47.4 ± 5.2	44.6 ± 5.9^#^	45.7 ± 5.4	46.7 ± 4.4
Placebo	45.8 ± 8.4	46.9 ± 9.8	47.3 ± 9.0	43.4 ± 7.1^#^	44.9 ± 8.5	41.8 ± 5.3^¥^
p-value*	0.476	0.939	0.980	0.628	0.760	0.007*
Thigh Girth (swelling) (% Δ from baseline)						
Green-lipped mussel oil blend	-	0.01 ± 0.02%	0.02 ± 0.02%	0.01 ± 0.02%	0.01 ± 0.02%	0.02 ± 0.04%
Placebo	-	0.00 ± 0.02%	0.02 ± 0.02%	0.01 ± 0.02%	0.02 ± 0.02%	0.02 ± 0.06%
p-value*	-	0.631	0.970	0.953	0.582	0.971
Maximum Voluntary Isometric torque (Strength) (Nm)						
Green-lipped mussel oil blend	72.8 ± 22.2	82.1 ± 19.7^#^	76.2 ± 19.8^#^	78.7 ± 21.5	79.9 ± 20.2	84.6 ± 22.4^¥,#^
Placebo	75.4 ± 19.3	82.1 ± 22.4^#^	74.9 ± 19.4^#^	74.6 ± 22.5	76.2 ± 20.8	83.9 ± 14.4
p-value*	0.721	0.843	0.987	0.569	0.608	0.872
%ΔQ_tw,pot_						
Green-lipped mussel oil blend	−27.8 ± 26.2	-	−30.4 ± 14.3	-	-	-
Placebo	−23.9 ± 24.0	-	−39.5 ± 24.3^¥^	-	-	-
p-value*	0.669	-	0.039	-	-	-

*, p-value between groups at distinct time points (p < 0.05 denotes statistical significance between groups; p > 0.05 denotes no statistical significance between groups); ¥, significantly different (p < 0.05) to pre- supplementation (baseline) within group; #, significantly different (p < 0.05) from previous time point within group. Pressure pain threshold and thigh girth (swelling) are expressed as % change (Δ) from the pre- supplementation (baseline) value within group, since baseline values were significantly different (p < 0.05) between groups. DOMS, delayed onset muscle soreness; % ΔQ_tw,pot_, % change in potentiated quadriceps twitch force from pre- to post-exercise (cycling time trial). Values expressed as mean ± SD.

The test of within-subject effects indicated that there was no significant effect (p > 0.05) of time on percent change from baseline in all post-supplementation time points for pressure pain threshold (PPT) values within the green-lipped mussel oil blend group. Post-hoc pairwise comparisons within the placebo group revealed a significant increase (p = 0.034; ∆, 0.12 ± 0.25%; 95% CI, 0.01 to 0.26%) in muscle tenderness at 24 h post-muscle damaging exercise only compared to before muscle damaging exercise in the placebo group (Table 3).

### Range of motion (knee flexion), swelling (thigh Girth) and isometric strength (torque)

The test of within-subject effects indicated that there was a significant effect (p < 0.01) of time on ROM within each group. Range of motion was significantly reduced (p < 0.05) at 48 h compared to 24 h post-muscle damaging exercise within the green-lipped mussel oil blend and placebo group. However, while no significant difference (p > 0.05) was found between groups for ROM values at baseline and post-supplementation (prior to, and at 24, 48 and 72 h post-muscle damaging exercise) a significant reduction (p < 0.05) in ROM occurred in the placebo group compared to the green-lipped mussel oil blend group at 96 h post-muscle damaging exercise (p = 0.007; ∆, −4.94 ± 8.10 degrees; 95% CI, −1.42 to −8.45 degrees) (Table 2). No significant difference (p > 0.05) was observed for the percent change from baseline in thigh girth (swelling) within or between groups at all-time points (Table 3).

The test of within-subject effects revealed that there was a significant effect (p < 0.01) of time on MVC (torque) within the both groups (Table 3). For both groups post- supplementation MVC significantly increased immediately prior to muscle damaging exercise compared to the baseline value (Placebo: p = 0.009; ∆, 6.68 ± 10.1 Nm; 95% CI, 1.30 to 12.10 Nm. Green-lipped mussel oil blend: p = 0.014; ∆, 9.41 ± 15.48 Nm; 95% CI, 1.16 to 17.66 Nm), and was significantly reduced at 24 h post-muscle damaging exercise compared to the MVC value obtained immediately prior to muscle damaging exercise (Placebo: p = 0.002; ∆, 7.13 ± 8.4 Nm; 95% CI, −2.65 to −11.61 Nm. Green-lipped mussel oil blend: p = 0.022, ∆, 6.02 + 11.00 Nm; 95% CI, −0.16 to −11.89 Nm). In addition, within the green-lipped mussel oil blend group only, MVC increased significantly at 96 h compared to 72 h post-muscle damaging exercise (p = 0.014; ∆, 4.67 ± 7.69 Nm; 95% CI, 0.57 to 8.77 Nm) and baseline (p = 0.003; ∆, 11.83 ± 15.24 Nm; 95% CI, 3.71 to 19.95 Nm). No significant difference (p > 0.05) in MVC was observed at any time point between groups.

### Quadriceps muscle fatigue

There was no significant difference (p > 0.05) in the percent change (%∆) in Q_tw, pot_ between groups at baseline, or within the green-lipped mussel oil blend group when comparing the %∆Q_tw,pot_ at baseline with 24 h following muscle damaging exercise. However, %∆Q_tw,pot_ was significantly greater at 24 h following muscle damaging exercise compared to baseline within the placebo group (p = 0.018 ∆, −11.4 ± 23.3%, 95% CI, −2.7 to −25.4%), and %∆Q_tw,pot_ was significantly greater for the placebo group compared with the green-lipped mussel oil blend group at 24 h following muscle damaging exercise (p = 0.039; ∆, −10.1 ± 24.7%; 95% CI, −4.3 to −29.1%) (Table 3), indicating greater muscle quadriceps fatigue in the placebo group.

### Skeletal and cardiac muscle damage blood markers

**Figure 1:**
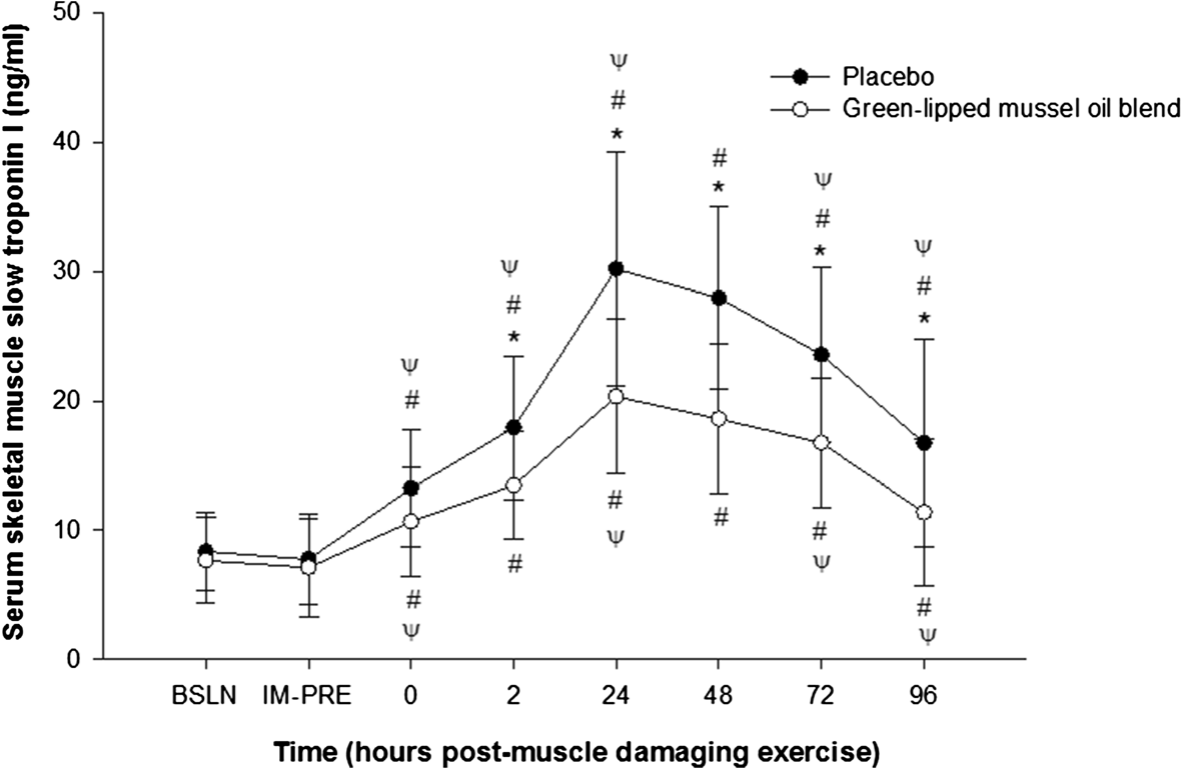
**Effect of supplementation on mean serum skeletal muscle slow troponin I concentration** (**ng**/**ml**) **pre**- **and post**-**eccentric exercise.** *, designates a statistical difference (p < 0.05) between groups at distinct time points. #, designates a significant difference (p < 0.05) compared to baseline (BSLN; pre-supplementation before eccentric exercise).ψ, designates a significant difference (p < 0.05) from previous time point within group. IM-PRE, immediately prior to eccentric exercise (post-supplementation). Data are expressed as mean ± SD.

Serum sTnI levels were not significantly different (p > 0.05) for baseline (pre- supplementation), immediately prior to, and immediately following (0 h) muscle damaging exercise (post-supplementation) either within or between groups (Figure 1). However, a significant increase (p < 0.05) in serum sTnI concentration, compared to baseline, was observed at 2, 24, 48, 72, and 96 h post- muscle damaging exercise within each group. Serum sTnI concentration peaked at 24 h following muscle damaging exercise, and compared to baseline increased by 260.2 ± 170.6% in the placebo group and by 165.1 ± 139.1% in the green-lipped mussel oil blend group. There was a significant reduction in the green-lipped mussel oil blend mean serum sTnI concentration compared to the placebo group at 2 h (p = 0.007; ∆, −4.5 ± 5.8 ng/ml; 95% CI, −0.9 to −8.0 ng/ml), 24 h (p < 0.001; ∆, −9.9 ± 10.1 ng/ml; 95% CI, −4.4 to −15.4 ng/ml), 48 hr. (p < 0.001, ∆, −9.4 ± 10.0 ng/ml; 95% CI, −4.7 to −14.0 ng/ml), 72 h (p = 0.003; ∆, −6.8 ± 9.1 ng/ml; 95% CI, −2.5 to −11.1 ng/ml), and 96 h (p = 0.02, ∆, −5.4 ± 10.5 ng/ml; 95% CI, −0.3 to −10.4 ng/ml) (Figure 1) following muscle damaging exercise.

**Figure 2:**
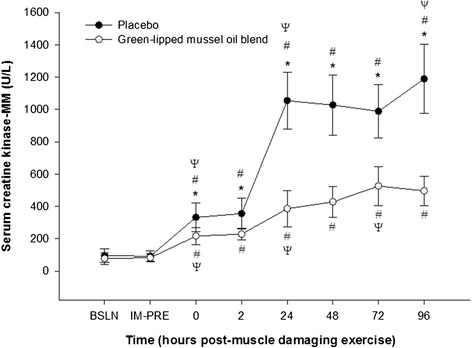
**Effect of supplementation on mean serum creatine kinase**-**MM concentration** **(ng/** **ml)** **pre-** **and post**-**eccentric exercise.** *, designates a statistical difference (p < 0.05) between groups at distinct time points. #, designates a significant difference (p < 0.05) compared to baseline (BSLN; pre-supplementation before eccentric exercise).ψ, designates a significant difference (p < 0.05) from previous time point within group. IM-PRE, immediately prior to eccentric exercise (post-supplementation). Data are expressed as mean ± SD.

Serum CK-MM levels were not significantly different (p > 0.05) for baseline, and immediately prior to muscle damaging exercise either within or between groups (Figure 2). However, a significant increase (p < 0.05) in serum CK-MM concentration, compared to baseline, was observed at 0, 2, 24, 48, 72, and 96 h post-muscle damaging exercise within each group. Serum CK-MM concentration peaked at 24 h following muscle damaging exercise for the placebo group and 72 h for the green-lipped mussel oil blend group, and compared to baseline increased by 1006.5 ± 631.2% in the placebo group and by 579.8 ± 287.4% in the green-lipped mussel oil blend group. A significant attenuation in the green- lipped mussel oil blend mean serum CK-MM concentration, compared to the placebo group, was detected at 0 h (p < 0.001; ∆, −116.1 ± 112.0 ng/ml; 95% CI, − 63.0 to −169.2 ng/ml), 2 h (p < 0.001; ∆, −127.3 + 101.7 ng/ml; 95% CI, −76.0 to −178.6 ng/ml), 24 hr. (p < 0.001; ∆, −386.1 ± 201.0 ng/ml; 95% CI, −562.6 to −775.6 ng.ml), 48 h (p < 0.001; ∆, −600.0 ± 208.5 ng/ml; 95% CI, −493.2 to −706.7 ng/ml), 72 h (p < 0.001; ∆, −463.7 ± 221.8 ng/ml; 95% CI, −360.0 to −568.0 ng/ml) and 96 h (p < 0.001; ∆, −693.0 ± 243.0 ng/ml; 95% CI, − 564.0 to −822.4 ng/ml) following muscle damaging exercise (Figure 2).

**Figure 3:**
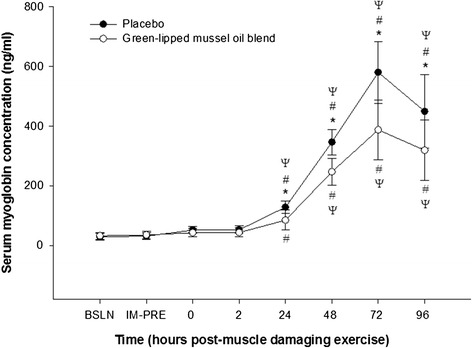
**Effect of supplementation on mean serum myoglobin concentration** **(ng/** **ml)** **pre-** **and post**-**eccentric exercise.** *, designates a statistical difference (p < 0.05) between groups at distinct time points. #, designates a significant difference (p < 0.05) compared to baseline (BSLN; pre-supplementation before eccentric exercise).ψ, designates a significant difference (p < 0.05) from previous time point within group. IM-PRE, immediately prior to eccentric exercise (post-supplementation). Data are expressed as mean ± SD.

Serum Mb levels were not significantly different (p > 0.05) when comparing baseline, and immediately prior to and at 0 h and 2 h muscle damaging eccentric exercise either within or between groups. However, a significant increase (p < 0.05) in serum Mb concentration, compared to baseline, was observed at 24, 48, 72, and 96 h post-muscle damaging exercise within each group. Serum Mb concentration peaked at 72 h following muscle damaging exercise (post-supplementation) for both groups, and compared to baseline increased by 1917.6 ± 876.3% in the placebo group and by 1109.5 ± 496.2% in the green-lipped mussel oil blend group. A significant attenuation in the green-lipped mussel oil blend mean serum Mb concentration compared to the placebo group was observed at 24 h (p < 0.001; ∆, −43.3 ± 37.1 ng/ml; 95% CI, −22.9 to −63.7 ng.ml), 48 hr. (p < 0.001; ∆, −99.4 ± 58.7 ng/ml; 95% CI, −67.7 to −131.2 ng/ml), 72 h (p < 0.001; ∆, −192.6 ± 159.8 ng/ml; 95% CI, −119.0 to −226.3 ng/ml) and 96 h (p = 0.001; ∆, −130.7 ± 161 ng/ml; 95% CI, − 49.1 to −212.2 ng/ml) following muscle damaging exercise (post-supplementation) (Figure 3). For serum cTnI and h-FABP concentration no significant differences (p > 0.05) were observed for all time points either within or between groups.

### Inflammatory and DNA oxidative stress markers

**Figure 4:**
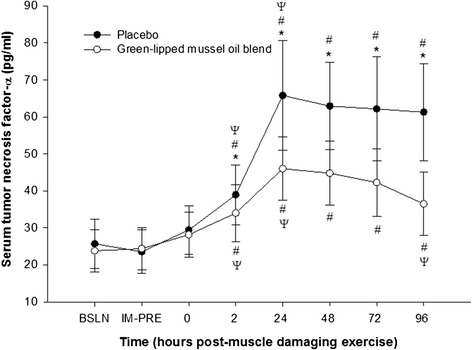
**Effect of supplementation on mean serum tumor**-**necrosis factor-** **α concentration** **(pg/** **ml)** **pre-** **and post**-**eccentric exercise.** *, designates a statistical difference (p < 0.05) between groups at distinct time points. #, designates a significant difference (p < 0.05) compared to baseline (BSLN; pre-supplementation before eccentric exercise).ψ, designates a significant difference (p < 0.05) from previous time point within group. IM-PRE, immediately prior to eccentric exercise (post-supplementation). Data are expressed as mean ± SD.

Mean serum TNF-α concentration did not significantly differ (p > 0.05) for baseline, immediately prior to, and at 0 h following muscle damaging exercise either within or between groups. However, a significant increase (p < 0.05) in serum TNF-α concentration, compared to baseline, was observed at 2, 24, 48, 72, and 96 h post-muscle damaging exercise within each group. Serum TNF-α levels peaked at 24 h following muscle damaging exercise, and compared to baseline increased by 156.2 ± 65.7% in the placebo group and by 93.3 ± 49.4% in the green-lipped mussel oil blend group. There was a significant reduction in mean serum TNF-α concentration for the green-lipped mussel oil blend group compared to the placebo group at 2 h (p = 0.042; ∆, −4.9 ± 8.1 pg/ml; 95% CI, −0.4 to −10.6 pg/ml), 24 h (p < 0.001; ∆, −19.8 ± 13.4 pg/ml; 95% CI, −11.1 to −28.5 pg/ml), 48 hr. (p < 0.001, ∆, −18.1 ± 12.7 pg/ml; 95% CI, −10.7 to −25.7 pg/ml), 72 h (p = 0.003; ∆, −19.9 ± 13.0 pg/ml; 95% CI, −11.3 to −28.5 pg/ml), and 96 h (p < 0.001, ∆, −24.8 ± 15.1 pg/ml; 95% CI, −16.8 to −32.8 pg/ml) following muscle damaging exercise (Figure 4).

Mean serum 8-OHdG concentration was not significantly changed (p > 0.05) either within or between groups for all time points.

### Nutrient intake and compliance

Mean daily nutrient intake, such as, for example, α-tocopherol, β-carotene, lycopene, vitamin C, magnesium, sodium, zinc, omega-3, total polyunsaturated fatty acids and saturated fatty acids did not differ significantly (p > 0.05) between groups during the course of the study. Compliance as estimated from return-capsule count was high (median, 99%).

## Discussion

The present study has shown that supplementing the diet of untrained men for 4 wk with a marine oil lipid and *n*-3 LC PUFA blend (PCSO-524®), derived from the New Zealand green lipped mussel (*P. canaliculus*), significantly reduced lower limb DOMS, quadriceps pain (tenderness), and peripheral muscle fatigue, and provided a protective effect against ROM (knee flexion) and isometric strength (torque) loss that can occur following downhill running designed to induce muscle damage. In addition, although blood markers of muscle damage and inflammation, sTnI, CK-MM, MB, and TNF-α, significantly increased following eccentric exercise in both groups, the rise in these blood markers were significantly suppressed on the green-lipped mussel oil blend supplemented diet compared to the placebo diet at most time points following muscle damaging exercise. No significant changes occurred between the green-lipped mussel oil blend and placebo group following eccentric exercise for swelling (thigh girth), and serum h-FABP, cTnI and 8-OHdG concentrations. Our findings may have implications for those who train regularly, especially since it has recently been shown that EPA and DHA levels (Omega-3 Index: percentage of EPA and DHA in total erythrocyte fatty acids) were low in a cohort of German elite winter endurance athletes [42], and importantly that *n*-3 LC-PUFA supplementation leads to a higher Omega-3 index level and decreased incidence of DOMS in healthy college aged individuals [43].

To date only two studies have been conducted in order to determine the efficacy of supplementation with this specific green-lipped mussel oil blend (PCSO-524®) on markers of EIMD and DOMS following muscle damaging [32] and exhaustive exercise [31]. While Baum et al. [31] found that 11 wk. of the green-lipped mussel oil blend supplemented diet reduced DOMS following an exhaustive 30 km run in male and female trained distance runners, Pumpa et al. [32] found no effect of 8 wk of supplementation with this specific green-lipped mussel oil blend on DOMS and functional and blood markers of EIMD following downhill running in trained men from a variety of sports. The divergent findings between the present study and the Pumpa et al. study [32] are difficult to reconcile, but is likely related to Pumpa and colleagues [32] using a lower dose (600 mg/day) of green-lipped mussel oil blend supplementation compared to the present study (1200 mg/day), and using a downhill running protocol of insufficient intensity to promote muscle damage and a robust inflammatory response in trained individuals.

### Effect of green-lipped mussel oil blend supplementation on delayed onset muscle soreness and pain threshold

We observed a significant decrease in DOMS at 96 h following muscle damaging exercise on the green-lipped mussel oil blend supplemented diet compared to the placebo diet, which is in agreement with some studies [15,17,19], but not all [14,21,32], that have shown that supplementing the diet with *n*-3 LC-PUFA prior to muscle damage attenuates DOMS following eccentric exercise and a 30 km run [31]. Delayed-onset muscle soreness appears many hours after muscle damaging exercise and peaks 24–72 h post-eccentric exercise [5], as was observed in both groups in the present study. What is clear is that while DOMS is not considered a disease or a disorder, it can limit further exercise in the days following an initial training bout [3].

In the present study the quadriceps pressure pain threshold (PPT) was used as an additional measure of muscle soreness in an attempt to ameliorate the subjective nature of the visual analog scale measure of soreness. We observed no change in the PPT for all time points within the green-lipped mussel oil blend group or between groups. However, in the placebo group perceived pain increased significantly 24 h following muscle damaging exercise compared to before muscle damaging exercise. These data seem to suggest that the green- lipped mussel oil blend supplemented diet may have afforded a protective effect against perceived pain developing in the quadriceps, and may be partially explained by the attenuation in muscle damage and the inflammatory response that occurred in this group [5]. While Tartibian et al. [19] have demonstrated that *n*-3 LC-PUFA supplementation for 30 days reduced perceived pain 48 hr. following eccentric exercise compared to placebo, other studies [16,21,32] have found no change in perceived pain following eccentric exercise when pre- treated with *n*-3 LC-PUFA.

### Effect of green-lipped mussel oil blend supplementation on range of motion (knee flexion), swelling (thigh Girth) and isometric strength (torque)

Among the numerous indirect markers of EIMD, muscle function measures such as muscle strength (MVC torque) and ROM are considered the best tools for quantifying muscle damage [2]. Eccentric-biased downhill running protocols typically generate 10-30% force loss after exercise, and that the reduction in MVC torque resulting from injury persists until the muscle function returns to its pre-injury condition (~24 h) [2]. Strength losses following eccentric exercise are likely due to the effects of muscle damage, and evidence suggests that excitation-contraction coupling failure plays a role [44]. In the present study there were no significant change observed for MVC torque at all-time points between groups or within the placebo group. However, MVC torque significantly increased compared to baseline (pre- supplementation) at 96 h following muscle damaging exercise on the green-lipped mussel oil blend supplemented diet. In support of this finding of an increase in muscle strength, Rajabi et al. [17] observed a significant increase in isotonic voluntary contractile strength of the quadriceps 24, 48 and 72 h following leg press eccentric exercise on a 30 day *n*-3 LC-PUFA supplemented diet compared to a placebo diet. Conversely, Gray et al. [14] observed no changes in MVC torque following 200 eccentric knee contractions, while Pumpa et al. [32] and Lenn et al. [21] observed no difference in muscle strength of the right and left quadriceps and non-dominant arm respectively between a placebo and *n*-3 LC-PUFA supplemented diet.

Many studies have documented decreases in the voluntary ROM (~20-45 degrees) following eccentric exercise, with full recovery not achieved until 10 days after exercise [44]. The mechanism to explain this decrease has been attributed to an increase in resting cytosol calcium levels, ultrastructure damage and/or an increase in fluid accumulation (swelling), and the measurement of joint ROM in muscle damage studies has been used as an indicator of passive muscle stiffness and soreness [45]. Our data indicate that muscle damaging exercise induced no loss of ROM in the green-lipped mussel oil blend group. However, within the placebo group ROM was significantly reduced (~4 deg) at 96 h post-muscle damaging exercise compared to baseline, and was significantly less (~4.9 deg) at 96 h following muscle damaging exercise on the placebo diet compared to the green-lipped mussel oil blend supplemented diet®. The protective effect provided by the *n*-3 LC-PUFA rich diet against ROM loss in the present study is similar to Tartibian et al. [19] and Rajabi et al. [17] who observed that on a *n*-3 LC-PUFA supplemented diet, compared to a placebo diet, the loss of knee ROM was significantly less post-eccentric bench stepping exercise and leg press eccentric exercise respectively, but are in contrast to the findings of Lenn et al. [21] and Phillips et al. [21] who observed no change in joint ROM following eccentric elbow flexion exercise.

Although swelling has been shown to occur following eccentric exercise and to be associated with the mechanisms of DOMS induced by eccentric exercise [46], there are studies that have shown a dissociation between when swelling and DOMS occur following eccentric exercise. Rodenburg et al. [47] have shown that MRI changes indicating the presence of edema do not to coincide with soreness following eccentric exercise (left forearm flexors) [47], while Clarkson et al. [35] noted that peak soreness occurred 2–3 d post-eccentric exercise (forearm flexor muscles) while peak swelling occurred 5 d following maximal effort eccentric actions of the forearm flexor muscles, while Yu et al. [48] has demonstrated that eccentric exercise (downstairs running) does induce muscle fiber swelling (soleus muscle), but it emerges at 7–8 d, and not at 2–3 d post-eccentric exercise when DOMS peaked. Based on the data from these studies [35,47,48] it is possible that we missed a potential effect of treatment on limb girth (swelling) since our final measurement of limb girth was at 96 h post- muscle damaging exercise.

### Effect of green-lipped mussel oil blend supplementation on quadriceps muscle fatigue

Potentiated quadriceps twitch force (Q_tw,pot_) assessed via magnetic stimulation before and after a 20-min cycling time trial pre- and post-supplementation (24 h following muscle damaging exercise) was used to quantify the degree of quadriceps muscle fatigue (∆Q_tw,pot_). The measurement of quadriceps twitch force produced by supramaximal magnetic stimulation of the femoral nerve has been shown to be a reliable method to detect quadriceps fatigue following loading [38,40,49]. This study has shown for the first time in humans that *n*-3 LC-PUFA supplementation provided a protective effect against the development of quadriceps muscle fatigue following a 20 min exhaustive cycling ergometer test compared to placebo supplementation. While the %∆Q_tw,pot_ was unaltered between pre- and post- supplementation (24 h following muscle damaging exercise) for the green-lipped mussel oil blend group, there was a significant decline (~65%) in the %∆Q_tw,pot_ post-supplementation compared to pre-supplementation for the placebo group. Our data are in agreement with animal studies [50,51] that have shown that rats fed fish oil, hindlimb skeletal muscle were more resistant to fatigue during continuous muscle twitch contractions, and recovered contractile force better between repeat bouts, compared to an *n*-6 LC-PUFA or saturated fat enriched diet.

### Effect of green-lipped mussel oil blend supplementation on blood markers of muscle damage, inflammation and DNA oxidative stress

Although a few studies have shown that eccentric exercise leads to myofibrillar remodeling specifically through Z-band related proteins, rather than muscle necrosis and inflammation [48,52], we have shown that supplementing the diet with a green-lipped mussel oil blend can mitigate the rise in a number of indirect markers of skeletal muscle damage and inflammation, and this effect persists for up to 96 h following muscle damaging exercise.

Slow skeletal troponin I is considered an early marker of EIMD, since it is particularly susceptible to calpain digestion, which may contribute to the early rise in plasma sTnI levels following eccentric exercise [53]. Our data are in agreement with Sorichter et al. [29] and Willoughby et al. [54] that a significant increase in serum sTnI can be detected within 2 h following muscle damaging exercise, and peaks within 24 h after the muscle injury- inducing sessions. In addition, we have shown for the first time that *n*-3 LC-PUFA supplementation can mitigate the increase in serum sTnI following muscle damaging exercise.

We observed a significant attenuation in serum Mb concentration in the green-lipped mussel oil blend group, compared to the placebo group, at 24, 48, 72 and 96 h following muscle damaging exercise, which is similar to the findings from a previous study [20] that showed that 30 d of *n*-3 LC-PUFA supplementation can moderate the rise in serum Mb at 24 and 48 h after eccentric exercise in untrained men. Myoglobin is an oxygen-binding heme protein found in skeletal and cardiac muscle, and thus is not specific for skeletal muscle, and h- FABP, which is involved in the transport and metabolism of fatty acids, is found in higher concentrations in the heart compared to human skeletal muscle [53]. However, both myoglobin and h-FABP have been proposed as useful markers of skeletal muscle injury in the absence of cardiac damage [30]. In the present study we did not observe any significant change in serum cTnI or h-FABP following muscle damaging exercise at any time point within either group, which suggests that the increase in serum myoglobin following muscle damaging exercise was likely the result of skeletal and not cardiac muscle damage.

Myofibrillar CK-MM is a cytosolic enzyme specifically bound to the myofibrillar M-line structure located in the sarcomere, and is also found in the space of the I-band sarcomeres where it provides support for muscle energy requirements. Whist we found that serum CK- MM significantly increased immediately following muscle damaging exercise and remained elevated in both groups for a further 96 h, there was a significant attenuation in serum CK- MM in the green-lipped mussel oil blend group, compared to the placebo group, for all-time points following muscle damaging exercise. A number of studies assessing the efficacy of *n*-3 LC-PUFA supplementation on EIMD and DOMS have not used serum CK-MM, but rather total serum CK concentration as an indirect marker of muscle damage. Given that total serum CK has not been shown to correlate with histological evidence of skeletal muscle damage [55] it is not surprising that considerable variability is observed in this blood marker among studies assessing the impact of *n*-3 LC-PUFA supplementation on muscle damage and DOMS following eccentric exercise [14,16,17,20,21,32].

An important aspect associated with the initiation and amplification of acute inflammation is the production of cytokines that are synthesized de nova by lymphocyte’s and monocytes at the site of muscle injury, and aid in directing inflammatory-related events [56]. At the onset of inflammation there is an upregulation of the pro-inflammatory cytokines interleukin-1β, and tumor-necrosis factor (TNF)-α. While IL-1β and TNF-α are most likely released by resident macrophages at the site of injury [56], and initiate the inflammatory response, TNF- α, in particular, has been shown to play a significant role in the muscle regeneration phase following muscle injury [57,58]. While we observed a significant increase in TNF-α for both groups following muscle damaging exercise, with serum TNF-α peaking at 24 h post-muscle damaging exercise, and remaining elevated for up to 96 h, the serum concentration of TNF-α was significantly lower in the green-lipped mussel oil blend group compared to the placebo group. At present the data are conflicting as to whether *n*-3 LC-PUFA can suppress the inflammatory response following eccentric exercise [16,20,21,32].

Oxidative stress-induced muscle damage has been shown to be associated with muscle soreness, and the resultant generation of free radicals causes oxidative damage to cellular DNA [59]. 8-hydroxy-2’ –deoxyguanosine (8-OHdG) is a product of oxidative DNA damage induced by the action of hydroxyl radicals on the DNA base deoxyguanosine (dG) and DNA single-strand breakage, and represents the most frequently used marker to assess DNA damage. Serum 8-OHdG has been shown to increase following eccentric isokinetic exercise in humans [59], but not after downhill running in rats [60] or humans [61]. In the present study we observed no significant increase in serum 8-OHdG following muscle damaging exercise in both groups compared to baseline, and no significant difference in serum 8-OHdG between groups at any time point. The lack of change in serum 8-OHdG following muscle damaging exercise does not exclude the possibility that changes in this marker were present in the active skeletal muscle, as the serum concentration 8-OHdG may be diluted in comparison with the actual site of generation (within muscle). In addition, the intensity/duration of the stimulus may not have been sufficient to overwhelm the body’s endogenous antioxidant system, and/or an upregulation of protective mechanisms against DNA damage developing [59], may have also played a role. It is surprising that serum 8- OhdG was not increased following muscle damaging exercise, especially given that structural protein within skeletal muscle was damaged (i.e. increase in serum sTnI). Changes in oxidative stress observed following muscle damaging exercise may not be the same when comparing blood and skeletal muscle, and thus we acknowledge that a limitation to our study is that we did not include additional biomarkers of oxidative stress.

While inflammation contributes to fibrosis, and causes pain and may well impair skeletal muscle function it does appear that inflammation represents a critical aspect of skeletal muscle repair and regeneration, and therefore blocking the inflammatory response with either pharmacological drugs or nutraceuticals may well hinder recovery [62]. With this in mind an important question that needs to be resolved is whether the most beneficial course of treatment should be to inhibit the inflammatory response or to let it progress naturally. Therefore, if there is a benefit in blocking inflammation, when is the appropriate time to do so and for how long post-muscle injury? While post-treatment of skeletal muscle injury is likely a more practical tactic, regardless of whether the injury is acute or slower to materialize such as with repetitive use injuries, it is not known at present whether *n*-3 LC-PUFA supplementation would be as effective in ameliorating EIMD and the inflammatory response if delivered post-injury only. This is an important question to answer since in some cases of skeletal muscle injury pre-treatment with anti-inflammatory agents for long periods of time is not always a realistic option for an individual.

### Anti-inflammatory mechanisms of action of PCSO-524®

In the present study the attenuation of a number of indirect markers of EIMD and inflammation cannot be explained entirely by the EPA and DHA content of PCSO-524®, since the total amount of EPA and DHA content consumed daily was 58 mg and 44 mg respectively, which are considerably lower amounts than previous studies [14-20] that have demonstrated a positive effect of *n*-3 LC-PUFA supplementation (0.3 - 2.0 g EPA/day and 0.2 – 1.0 g DHA/day) on mitigating EIMD, DOMS and inflammation. It has been shown the green-lipped mussel oil blend used in the present study, which contains up to 91 fatty acid components [23], has more potent anti-inflammatory activity than fish oil, which contains abundant EPA, in various animal models of arthritis, and inflammatory bowel disease [22]. Therefore, it is possible that additional constituents of the green-lipped mussel oil blend, which may act synergistically with the *n*-3 LC-PUFA, may also be partially responsible for its anti-inflammatory effects. The green-lipped mussel oil blend contains polyphenols (oleuropein and hydroxtyrosol) and oleic acid, which are anti-inflammatory, and postulated to reduce risk factors for heart disease, lower cancer mortality, and reduce inflammation [22,63]. It has been shown that furan fatty acids, which are a minor component of the green- lipped mussel oil blend, exhibit more potent anti-inflammatory activity than EPA in a rat model of adjuvant-induced arthritis [25], and which possess potent free-radical scavenging abilities [64], may explain, at least in part, why in the present study the green-lipped mussel oil blend was effective in attenuating EIMD, DOMS and inflammation, given the very low dose of EPA and DHA.

## Conclusion

In conclusion, the present study has shown that supplementing the diet of untrained men for 30 days with 1200 mg/d of a marine oil lipid and *n*-3 LC PUFA blend (PCSO-524®), derived from the New Zealand green lipped mussel, attenuated indirect markers of muscle damage and inflammation following downhill running designed to induce muscle damage, and may represent a useful therapeutic agent for mitigating muscle damage and inflammation following unaccustomed and/or eccentric exercise.
